# Investigation of toll-like receptor (TLR) 4 inhibitor TAK-242 as a new potential anti-rheumatoid arthritis drug

**DOI:** 10.1186/s13075-020-2097-2

**Published:** 2020-01-23

**Authors:** Snigdha Samarpita, Joo Young Kim, Mahaboob Khan Rasool, Kyoung Soo Kim

**Affiliations:** 1 Immunopathology Lab, School of Bio Sciences and Technology, Vellore Institute of Technology (VIT), Vellore, Tamil Nadu 632014 India; 2 Department of Clinical Pharmacology and Therapeutics, Kyung Hee University School of Medicine, Kyunghee-daero 23, Dongdaemun-gu, Seoul, 02447 South Korea; 3 East-West Bone and Joint Disease Research Institute, Kyung Hee University Hospital at Gangdong, Gandong-gu, Seoul, 05278 South Korea

**Keywords:** TLR4 inhibitor, TAK-242 (resatorvid), Rheumatoid arthritis (RA), Damage-associated molecular patterns (DAMPs), Lipopolysaccharide (LPS), Poly(I:C), Adjuvant-induced arthritis (AIA) rat model, Fibroblast-like synoviocytes (FLS)

## Abstract

**Background:**

Proper blocking of toll-like receptor (TLR) activation during disease progression has been reported to have inhibitory effect on the pathogenesis of rheumatoid arthritis (RA). We tested whether the TLR4 inhibitor TAK-242 had potential as a remedy for rheumatoid arthritis.

**Methods:**

The therapeutic effect of TAK-242 was tested in vitro using the human rheumatoid fibroblast-like synoviocyte (FLS) line MH7A or primary human FLS and in an adjuvant-induced arthritis (AIA) rat model.

**Results:**

TAK-242 dose dependently inhibited the increased expression of IL-6, IL-8, MMP-1, and VEGF in LPS-stimulated MH7A cells. It also inhibited the expression of IL-6 and IL-8 in poly(I:C), TLR3 activator-stimulated primary FLS, but not in IL-1β-stimulated primary FLS. These findings suggest that TAK-242 blocks a specific signaling pathway to some degree. Further, TAK-242 slightly inhibited mobilization of NF-κB into nuclei. In the AIA rat model, TAK-242 significantly reversed the body weight and paw thickness of AIA rats to the normal state at a dose of 5 mg/kg, but not at 3 mg/kg, and reduced the increased serum level of IL-6 and VEGF in AIA rats. It also significantly ameliorated inflammatory symptoms of joint tissues at day 21 of treatment, according to histology and RT-PCR.

**Conclusions:**

Based on the drug repositioning concept, TAK-242, which is used for the treatment of TLR4-mediated inflammatory diseases, shows potential for cost-effective development as a remedy for rheumatoid arthritis or to control the progression of RA.

## Introduction

Tissue and cells are injured during chronic and persistent inflammation of rheumatoid arthritis (RA). Endogenous molecules such as nuclear proteins, RNA, and DNA may be released into synovial fluid and then activate inflammatory responses through the signaling pathway of toll-like receptors (TLRs) expressed in cells such as macrophages, synovial cells, and preosteoclasts located in joints [1]. Although TLRs are the front line of innate immunity, they have been known to play an important role in triggering immune responses by recognizing various molecules released from damaged cells known as damage-associated molecular patterns (DAMPs) as well as pathogen-associated molecular patterns (PAMPs) such as bacterial lipopolysaccharides (LPS), viral RNA, CpG-containing DNA, and flagellin [2, 3]. The specific bindings of PAMPs and DAMPs to TLRs may trigger main adaptor protein MyD88-dependent or independent pathways [4]. All these inflammatory responses through TLRs signaling pathways remove harmful stimuli or repair damaged tissues. However, such endogenous damage signals can induce an inappropriate innate response, resulting in harmful inflammation that can lead to more destruction than the original injury [5, 6].

The relevance of TLRs to RA has been investigated through experimental models. TLR2 and TLR4 have been extensively studied, and TLR5 and TLR7 have recently gained attention in RA pathology [1]. TLR4 is mainly expressed in monocytes, macrophages, granulocytes, and the spleen, but the additional expression of TLR4 in chondrocytes, osteoblasts, and synoviocytes implicates TLR4 in the pathophysiology of the musculoskeletal system [7]. Thus, TLR4 appears to be more important than other TLRs in RA [8]. Accordingly, proper blocking of TLR4 activation during disease progression may help control RA.

TLR4 antagonists have been developed to beneficially block TLR4 signaling in various diseases such as sepsis, septic shock, lung inflammation, and RA [4]. TLR4 antagonists, anti-TLR4 antibodies, decoy peptides, and small molecule inhibitors inhibit the interaction of ligands with TLR4 to block triggering of the TLR4 signaling pathway in an extracellular or intracellular manner [4, 9]. In particular, cell-permeable TAK-242 (resatorvid) selectively binds to TLR4 and interferes with interactions between TLR4 and two adaptor molecules of TLR4, toll/interleukin-1 receptor domain-containing adaptor protein (TIRAP), or toll/interleukin-1 receptor domain-containing adaptor protein inducing interferon-β-related adaptor molecule (TRAM) [10]. TAK-242 is a small molecule suppressor of pathogen-induced release of inflammatory cytokines and acts by inhibiting TLR-4-mediated signaling. It also shows inhibitory effects on the production of nitric oxide (NO) or tumor necrosis factor (TNF)-alpha induced by the TLR4-specific ligand LPS [11]. Because of its suppression of cytokine levels, TAK-242 is known as an effective treatment for severe sepsis and may be a new therapeutic agent for other inflammatory diseases. Under the concept of drug repositioning, we wanted to determine the potential for TAK-242 to have a therapeutic effect against RA. In this study, we show that TAK-242 inhibits the expression of inflammatory cytokines in LPS-stimulated FLS and demonstrate anti-arthritic effects in a complete Freund’s adjuvant (CFA)-induced arthritis (AIA) rat model.

## Materials and methods

### Cells and culture

All in vitro experiments were carried out with primary fibroblast-like synoviocytes (FLS) or the FLS-transformed cell line MH7A [12]. Primary FLS were purchased from Cell Applications Inc. (San Diego, CA). MH7A was obtained from Dr. Lee SY at Gyeongsang University Hospital. After the cells had grown to confluence, they were split at a 1:4 ratio. Primary FLS passages 3 to 6 were used for all experiments.

### Quantitative polymerase chain reaction

Quantitative RT-PCR (qRT-PCR) was then carried out using the EvaGreen Supermix PCR kit (Bio-Rad, Hercules, CA, USA) on a thermal cycler (Bio-Rad, Hercules, CA). The primer pairs of NF-κB-p65 and AP-1 were designed using NCBI Primer 3 blast; primer sequences are as follows: NF-κB-p65, forward 5′-CTC ACC GGC CTC ATC CAC AT-3′, reverse 5′-TGG CTA ATG GCT TGC TCC AG-3′; AP-1, forward 5′-GAC TGC AAA GAT GGA AAC GAC C-3′, reverse 5′-AGA AGG TCC GAG TTC TTG GC-3′. qRT-PCR was performed in triplicate for each sample, and the relative mRNA expression levels of target genes were normalized to GAPDH (forward 5′-AGG TCG GTG TGA ACG GAT TTG-3′, reverse 5′-TGT AGA CCA TGT AGT TGA GGT CA-3′) using the 2-^ΔΔCt^ method as described previously [13].

### Western blot analysis

As described previously [14], cell lysates of LPS-stimulated MH7A were then probed with various rabbit polyclonal antibodies for p-ERK1/2, p-P38, p-Jun N-terminal kinase (JNK), and GAPDH (Cell Signaling Technology, Beverly, MA, USA). Nuclear extracts were prepared by cell lysis followed by nuclear lysis as described previously [15]. Levels of NF-κB and AP-1 in the nuclei were measured by western blot using anti-NF-κB-p65 and anti-AP-1 antibodies (Santa Cruz Biotechnology).

### Cytokine quantification by ELISA

Conditioned media were collected 24 or 48 h later. Briefly, culture supernatants were centrifuged and the supernatants were collected and analyzed for IL-6, IL-8, MMP-1, and VEGF with an ELISA kit (R&D Systems, Minneapolis, MN, USA). Serum levels of proinflammatory cytokines IL-6 and VEGF were quantified in serum samples using specific rat ELISA kits (PeproTech, NJ, USA) according to the manufacturer’s recommendations.

### Immunofluorescence staining

Human primary FLS were stimulated with LPS at 2 μg/ml or IL-1β at 10 ng/ml for 90 min in order to visualize the translocation of NF-κB to the nucleus, as described previously [15]. The cells were incubated with 5 μg/ml rabbit polyclonal anti-NF-κB-p65 antibody (Santa Cruz Biotechnology) overnight, followed by counterstaining with 0.1 mg/ml DAPI. The slides were visualized using confocal microscopy (Carl Zeiss, Oberkochen, Germany).

### Experimental animals and design

Wistar albino female rats (180–200 g) were procured from the animal unit facility of School of Bio Sciences and Technology (SBST), Vellore Institute of Technology (VIT, Vellore, India). Prior to the experiments, the rats were acclimated to housing conditions maintained at 12 h light/dark cycle, a temperature of 25 ± 2 °C, and a properly humid environment. They were fed with standard rodent diet and water ad libitum. All experimental procedures were conducted in compliance with the norms of the Committee for the Purpose of Control and Supervision on Experiments on Animals (CPCSEA), India. All experimental setups were certified by the Institutional Animal Ethical Committee (IAEC). Experimental rats were randomized into five groups (six in each group), and arthritis was induced as described previously [16]. The study lasted 21 days, wherein all treatments of TAK-242 (Cayman Chemical, Ann Arbor, MI) were carried out intraperitoneally from day 11 to day 20 post-CFA induction on day 0.

### Assessment of physical symptoms in arthritis

In order to assess the severity of arthritis, paw thickness and body weight were measured on day 0 and on every third day following complete Freund’s adjuvant (CFA) induction using Vernier calipers and a weighing balance, respectively. Though plethysmometer technique for measuring paw volume is considered more advanced, the Vernier caliper method is still considered as the most sensitive and gold standard method of assessing the anti-inflammatory activity. Evaluation of arthritis severity was also assessed daily by visual observation. Additionally, radiograph images of the hind limbs were taken in order to assess the presence of joint deformity. All radiographs were taken using an MBR-1505R (Hitachi Medical Corporation, Japan) with 0.5 mm focal spot and processed with X-ray film (Kodak Diagnostic Film) placed 60 cm below the X-ray source with 1 s exposure at 5 mA and 40 kV. Arthritis severity was evaluated in a blinded manner by a certified radiologist on the basis of radiographic changes in the joints including joint space, soft tissue volume, and degenerative joint changes.

### Histologic analysis

Following sacrifice, the rat left hind limbs were dissected and preserved in 10% formalin for histological assessment. As described previously [17], the sections were then deparaffinized, mounted, and stained with hematoxylin-eosin for visualization under an inverted microscope equipped with digital cameras (Olympus photomicroscope, Tokyo, Japan) for arthritis scoring in terms of cellular infiltration, joint space, pannus formation, and bone erosion. For immunohistochemical studies as described previously [18], sections were further immunostained with primary anti-NF-κB-p65 (Cell Signaling Technology, Beverly, MA, USA) and anti-AP-1(Cell Signaling Technology, Beverly, MA, USA) antibodies. Immunohistochemical scoring was carried out for NF-κB-p65 and AP-1 separately on the basis of a semi-quantitative scale and finally combined into a total score. Images were photographed randomly, and staining intensity was evaluated by an experienced pathologist on a scale of 1–4 (0 = absent, 1 = weak, 2 = moderate, 3 = high, and 4 = very high) in a blinded fashion. The sum of the scores of three independent sections of the knee joint per rat from each experimental group was averaged and plotted on a bar group.

### Statistical analysis

Experimental data are expressed as mean ± standard error of the mean (SEM). Differences between groups were analyzed using the nonparametric Kruskal-Wallis test. If a statistical difference was detected (*P* < 0.05), post hoc pairwise group comparisons were performed using Dunn’s test with Bonferroni’s multiple-testing correction. Prism software v.5 (Graphpad Software, San Diego, CA) was used for statistical analysis and graphing. Differences were considered statistically significant at *P* < 0.05.

## Results

### TAK-242 inhibits the increased expression of IL-6, IL-8, MMP-1, and VEGF in LPS-stimulated fibroblast-like synoviocytes in vitro

**Fig. 1:**
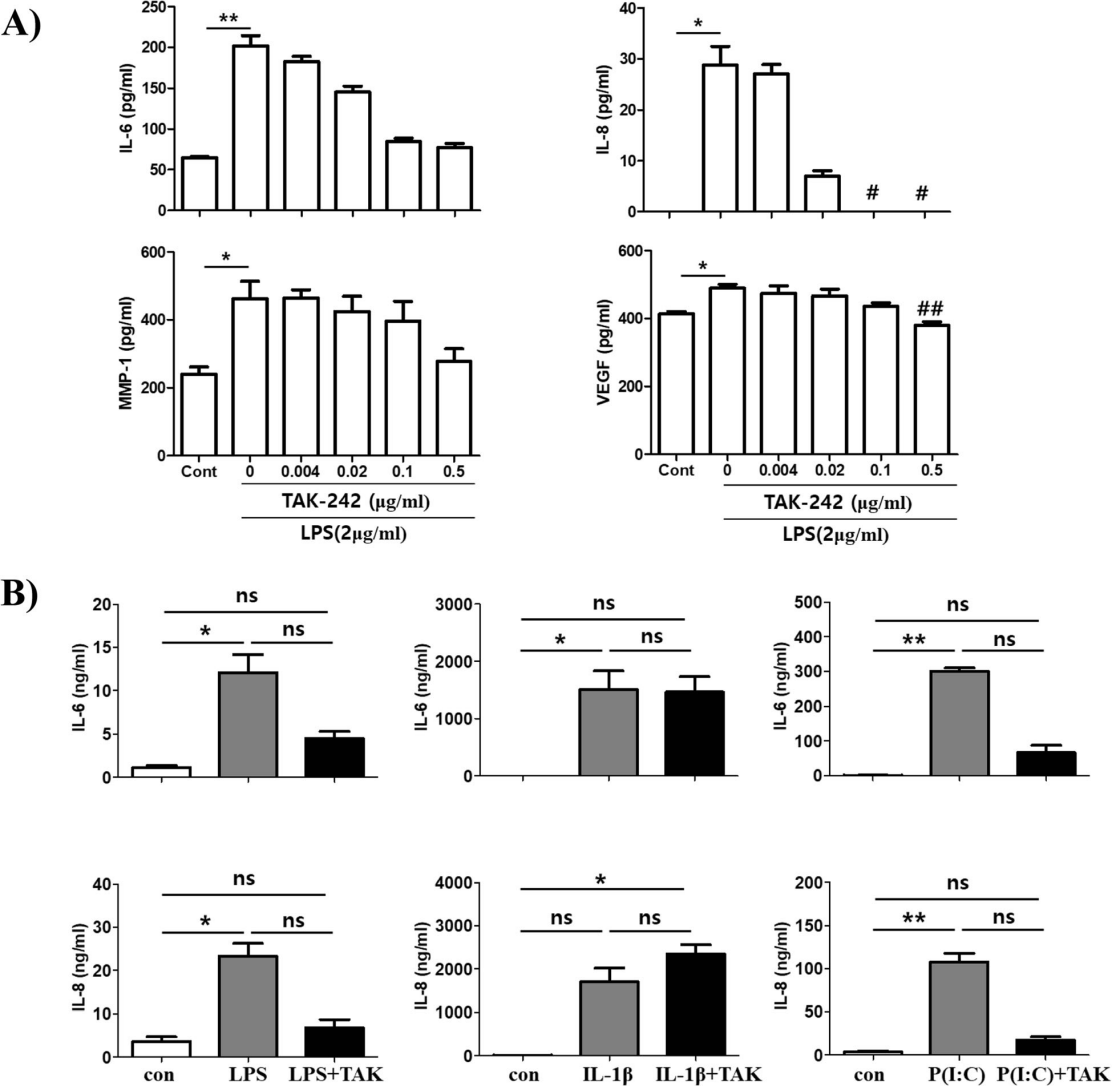
Inhibitory effects of TAK-242 on the production of pro-inflammatory mediators. **a** Human fibroblast-like synoviocyte (FLS) cell line MH7A cells (2.5 × 10^5^ cells) were cultured overnight in 6-well plates containing 2 ml of media and then treated with LPS (2 μg/ml) and various concentrations of TAK-242 for 24 h. **b** Human primary FLS cells (2.5 × 10^5^ cells) were cultured overnight in 6-well plates containing 2 ml of media. The cells were treated with LPS (2 μg/ml), IL-1β (10 ng/ml), or poly(I:C) (30 μg/ml) in the presence or absence of TAK-242 (0.5 μg/ml) for 24 h. The expression levels of IL-6, IL-8, MMP-1, and VEGF were checked by ELISA from culture supernatants. The data shown are representative of three independent experiments, and similar results were obtained from all three. Differences between groups were analyzed using the nonparametric Kruskal-Wallis test. If a statistical difference was detected (*P* < 0.05), post hoc pairwise group comparisons were performed using Dunn’s test with Bonferroni’s multiple-testing correction. Only significant difference between groups was indicated with “*” or “#.” Values are expressed as mean ± standard error of the mean. *P < 0.05, **P < 0.01 versus control group, ^#^*P* < 0.05, ^##^*P* < 0.01 versus TAK-242 non-treated group. P(I:C), poly(I:C)

First, to estimate if LPS stimulates pro-inflammatory mediators in human FLS, MH7A cells were treated with LPS at 2 μg/ml for 24 h. Treatment with the TLR4 inhibitor TAK-242 slightly inhibited the expression of IL-6 and MMP-1 at 0.02 μg/ml, but it was not significant. However, IL-8 and VEGF expression was significantly inhibited at 0.5 μg/ml in LPS-stimulated MH7A cells (Fig. 1a). Next, primary human FLS were stimulated with LPS, IL-1β, or poly(I:C) in combination with TAK-242. As shown in Fig. 1b, TAK-242 treatment showed the trend of inhibiting the increased expression of IL-6 and IL-8 caused by LPS or poly(I:C), but did not alter IL-1β-stimulated FLS. These findings suggest that TAK-242 treatment effectively inhibited the signaling pathway through TLR4 and TLR3 activation, but not through the IL-1β receptor.

### TAK-242 exerts an anti-inflammatory effect by inhibiting the mobilization of NF-κB and AP-1 into the nucleus in LPS-stimulated FLS

**Fig. 2:**
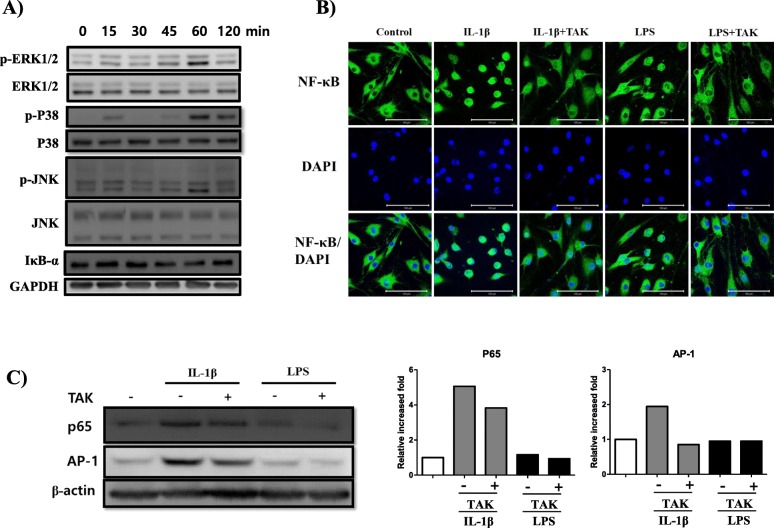
Stimulation of signaling pathways by LPS and inhibition by TAK-242 in synovial fibroblasts. **a** Time course of signaling pathways activated by LPS. **b** Confocal image of NF-κB location in the nucleus and cytoplasm. TAK-242 inhibited the migration of NF-κB into the nucleus from the cytoplasm. **c** Western blot of nuclear protein. Protein levels of NF-κB and AP-1 in the nucleus were detected by western blot. The increased levels of NF-κB-p65 and AP-1 in response to IL-1β (10 ng/ml) or LPS (2 μg/ml) were weakly inhibited by TAK-242. The band density was measured by ImageJ program. The data shown are representative of three independent experiments, and similar results were obtained from all three

To confirm which signaling pathways LPS stimulates, the signaling pathways of MAP kinase and IκB-α kinase was investigated in LPS-stimulated synovial fibroblast MH7A cells (Fig. 2a). LPS slightly stimulated the MAP kinase signaling pathway such as ERK1/2, P38, and JNK after 60 min of stimulation. In addition, it did stimulate the signaling pathway through IκB-α kinase. Consistent with the activation of signaling pathways in response to LPS, LPS mobilized NF-κB into the nuclei of primary human FLS, although the response was much weaker than with IL-1β stimulation (Fig. 2b). TAK-242 treatment slightly inhibited the mobilization of NF-κB into nuclei based on confocal imaging. In addition, IL-1β-mobilized NF-κB seemed to be partly inhibited by TAK-242 in confocal images. The TAK-242-mediated inhibition of mobilization was further confirmed by western blot using nuclear proteins (Fig. 2c). The levels of p65 and AP-1 in the nuclei were not significantly increased by stimulation with LPS, but even the slightly increased levels were mitigated by TAK-242 treatment. In contrast, the levels of p65 and AP-1 increased greatly in the nuclei in response to IL-1β, but were partly inhibited by TAK-242 treatment, suggesting that TAK-242 partly inhibited the activation of NF-κB and AP-1.

### TAK-242 recovers decreased body weight and paw thickness in adjuvant-induced arthritis rats

**Fig. 3:**
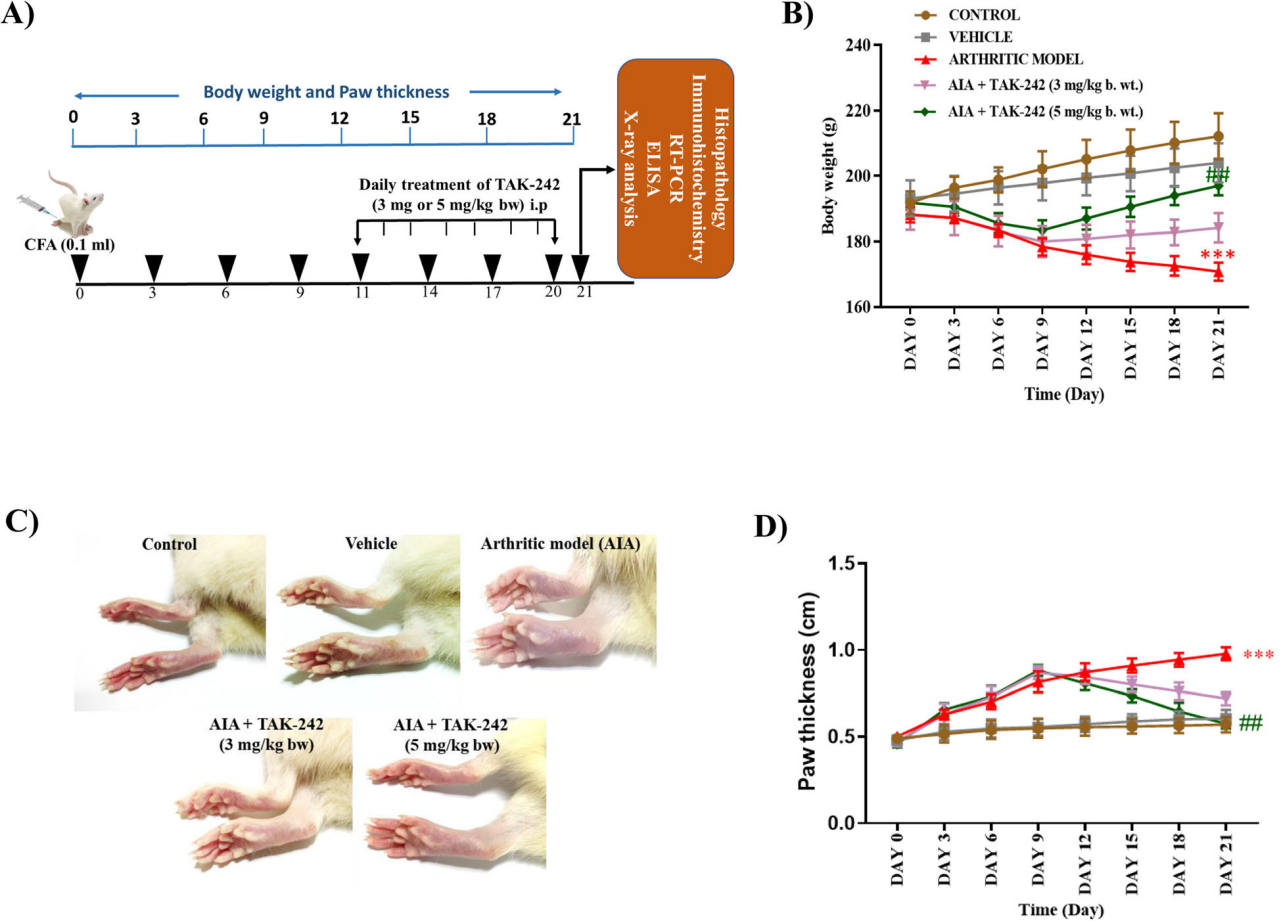
Therapeutic effects of TAK-242 on physical symptoms in adjuvant-induced arthritis (AIA) rats. **a** In vivo experimental design and schedule. Wistar albino rats were immunized with complete Freud’s adjuvant (CFA) for AIA on day 0. On day 11, the vehicle group of rats was treated with vehicle alone (0.1% DMSO) and adjuvant-induced arthritic (AIA) rats were intraperitoneally (i.p.) treated with TAK-242 (3 mg or 5 mg of 0.1% DMSO/kg) daily until the 20th day. **b** Changes in body weight upon CFA immunization and TAK-242 treatment. TAK-242 treatment (5 mg/kg) significantly recovered the decreased body weight of AIA rats. **c** Macroscopic images of a representative rat displaying the gross features of paw joints on day 21. **d** Measurement of paw thickness during the experimental period. Values are expressed as mean ± standard error of the mean. ***P < 0.001 versus control group, ^##^*P* < 0.01 versus arthritis model

To demonstrate the anti-arthritic effect of TAK-242 in an arthritis animal model, AIA rats induced by injection of complete Freund’s adjuvant (CFA) into the foot pad of the hind paw were treated daily for 10 days with TAK-242 at a dose of 3 mg or 5 mg/kg of body weight from 11 days after arthritis induction according to the schedule shown in Fig. 3a. Changes in body weight and paw thickness were measured every 3 days, as shown in Fig. 3b, d. The arthritic rats exhibited marked loss in body weight over the first weeks after arthritic induction followed by a deliberate change in body weight in the subsequent weeks. Conversely, arthritic rats showed a remarkable gain in body weight following treatment with TAK-242 (5 mg/kg), and a minimal increase in body weight was observed in arthritic rats treated with TAK-242 (3 mg/kg). Correspondingly, the increased paw volume of arthritic rats was highly comparable to that of the normal control rats, which showed no changes, while a significant reduction in paw volume was seen in arthritic rats treated with TAK-242 (5 mg/kg) (Fig. 3c, d).

### TAK-242 ameliorates local joint inflammation and bone damage in AIA rats

**Fig. 4:**
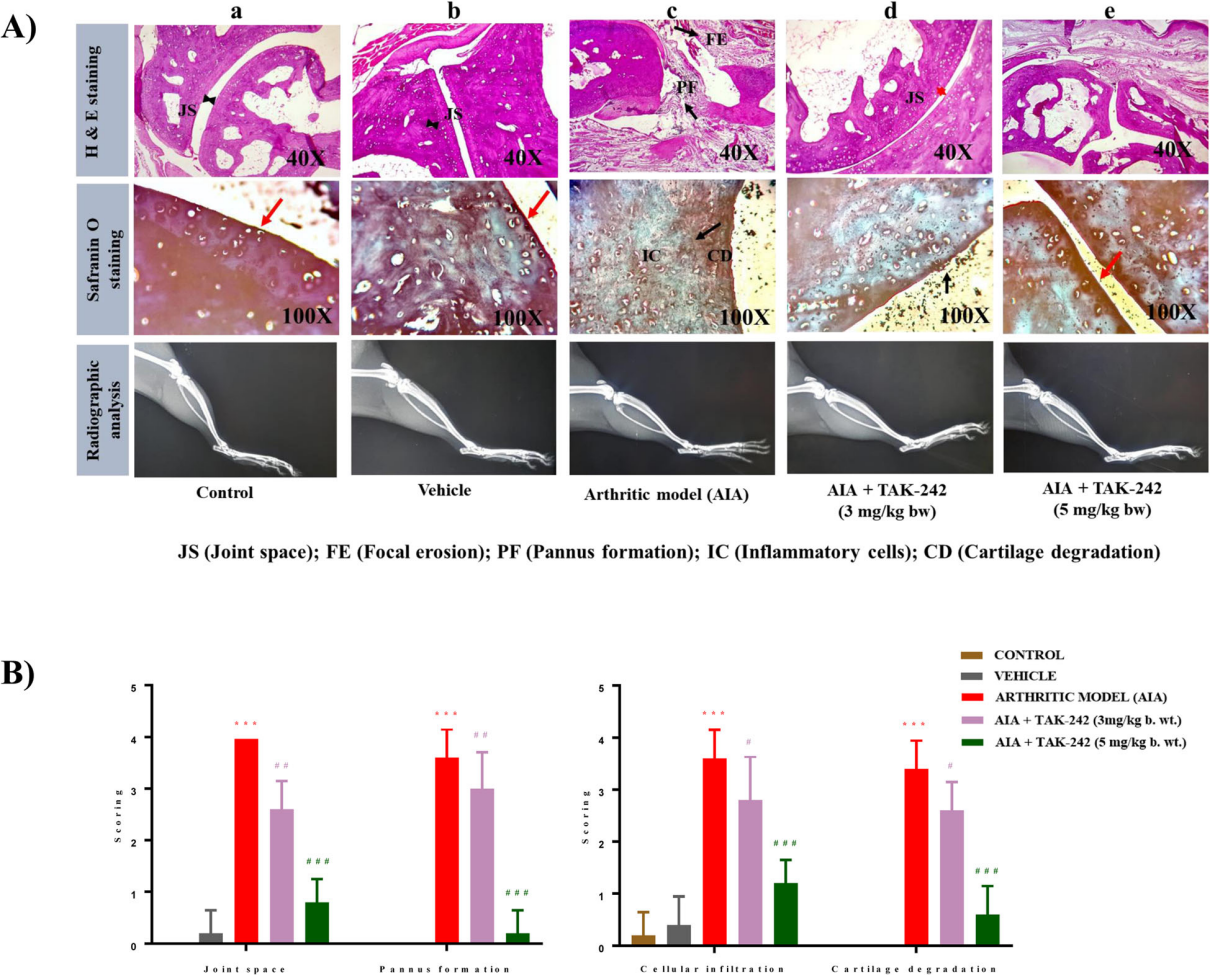
Histologic and radiographic analysis of joint tissues of AIA rats. **A** Images of H&E staining, Safranin O staining, and radiography of joints in (a) control rats, (b) control rats treated with vehicle, (c) AIA rats, (d) AIA rats treated with TAK-242 (3 mg/kg), and (e) AIA rats treated with TAK-242 (5 mg/kg). In H&E staining, the AIA group showed pannus formation (PF) and focal erosion (FE). TAK-242 treatment (5 mg/kg) exhibited normal JS and resolved PF and FE. Consistent with H&E staining data, Safranin O staining showed that the AIA group had extreme cartilage degradation; TAK-242 treatment (5 mg/kg) inhibited the extreme cartilage degradation (red arrow). In contrast, TAK-242 treatment at a dose of 3 mg/kg did not affect the reduced joint space (red arrow) and cartilage degradation (black arrow). Radiographic images of ankle and knee joints from representative rats on day 21 show no differences between groups. **B** The graph denotes the mean score of joint space, pannus formation, cellular infiltration, and cartilage degradation based on a 4-point scale (0 = normal, 1 = mild, 2 = moderate, 3 = high, and 4 = extreme). ***P < 0.001 compared with control. ^#^*P* < 0.05, ^##^*P* < 0.01, ^###^*P* < 0.001 compared with arthritic model

To evaluate the effect of TAK-242 in inhibiting joint damage, knee joints were stained with hematoxylin-eosin (H&E) and Safranin O staining for assessment of cartilage damage. Histopathological assessment showed areas of cartilage damage, cellular infiltration, pannus formation, and joint space in the knee joints of experimental rats. Histopathology images of control rats revealed no cartilage destruction and the absence of pannus formation with few infiltrated cells (Fig. 4A). Conversely, massive cellular infiltration, pannus formation, and severe cartilage damage were observed in arthritic rats with a maximum score of 4. In contrast to these severe pathological changes, rats treated with TAK-242 at 5 mg/kg showed minimal cellular infiltration with a score of 1 and no remarkable cartilage damage or pannus formation. However, no such changes were seen in arthritic rats treated with TAK-242 at 3 mg/kg as compared to adjuvant-induced arthritic rats. Meanwhile, radiography of hind limbs of the arthritic rats showed narrowing of joint spaces with irregular bone erosion compared to that of normal control rats. However, the radiographic findings manifested no such typical changes in arthritic rats treated with TAK-242 (5 mg/kg). As shown in Fig. 4B, the histological evaluation of joint space, pannus formation, cellular infiltration, and cartilage degradation was scored on a semi-quantitative scale of 1–4.

### TAK-242 significantly reduces the expression of important transcription factors in the synovial tissue of AIA rats

**Fig. 5:**
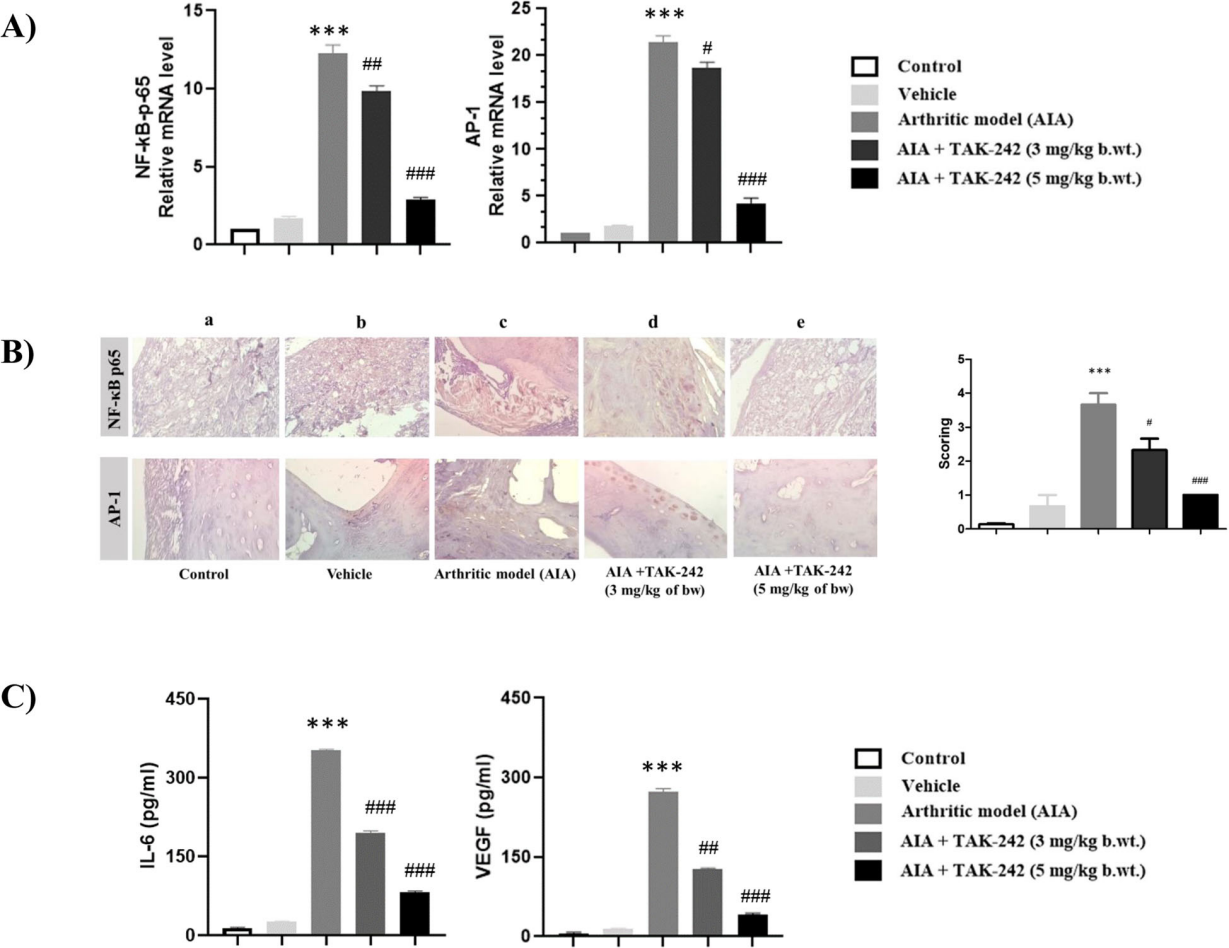
Inhibitory effects of TAK-242 on the expression levels of inflammatory transcription factors (NF-κB-p65 and AP-1) in the synovial tissues of AIA rats. **A** The mRNA expression levels of NF-κB-p65 and AP-1 were determined by real-time PCR. Increased levels of NF-κB-p65 and AP-1 mRNA were significantly reduced by TAK-242 treatment. **B** Immunohistochemical analysis of NF-κB-p65 and AP-1 was carried out on tissue sections excised from (a) control rats, (b) control rats treated with vehicle, (c) AIA rats, (d) AIA rats treated with TAK-242 (3 mg/kg), and (e) AIA rats treated with TAK-242 (5 mg/kg). Scoring was also determined by staining intensity of NF-κB-p65 and AP-1 and finally combined into a total score. Magnification × 40. **C** Serum concentrations of IL-6 and VEGF were determined by ELISA. Values are expressed as mean ± standard error of the mean. ***P < 0.001 compared with control. ^#^*P* < 0.05, ^##^*P* < 0.01, ^###^*P* < 0.001 compared with arthritic model

In order to estimate the underlying molecular mechanisms of TAK-242 in disease remission, we estimated mRNA expression levels of inflammatory transcription factors NF-κB-p65 and AP-1 in the synovial tissues of experimental rats by qRT-PCR. We observed a significant increase in the mRNA expression levels of both NF-κB-p65 and AP-1 in the synovial tissue of arthritic rats when compared to control rats (Fig. 5A). However, treatment of rats with established clinical signs of arthritis with TAK-242 at 5 mg/kg resulted in a highly significant reduction in mRNA expression levels of NF-κB-p65 and AP-1 when compared to arthritic rats and rats treated with TAK-242 at 3 mg/kg. To further evaluate the effect of the therapeutic intervention of TAK-242 on the protein expression of NF-κB-p65 and AP-1, we performed immunohistochemical analysis of synovial tissue sections. Tissue sections from arthritic rats showed an increase in the expression of NF-κB-p-65 and AP-1 compared to the normal control group (Fig. 5B). Consistent with mRNA levels, a significant reduction in protein expression was observed in immunohistochemical sections from arthritic rats treated with TAK-242 at 5 mg/kg. However, the protein expression was not significantly comparable in arthritic rats treated with TAK-242 at 3 mg/kg.

### TAK-242 diminishes serum IL-6 and VEGF levels in adjuvant-induced arthritic rats

To evaluate the anti-inflammatory effect of TAK-242 on circulating levels of pro-inflammatory cytokines, we quantified the serum levels of pro-inflammatory cytokines IL-6 and VEGF by ELISA in experimental rats. Serum levels of both IL-6 and VEGF were significantly increased in arthritic rats as compared with control rats (Fig. 5C). On the other hand, arthritic rats treated with TAK-242 showed substantial reduction in the levels of IL-6 and VEGF to concentrations almost similar to the serum of normal control rats.

## Discussion

Many therapeutic agents have been developed to treat or control the symptoms of rheumatoid arthritis (RA). In particular, therapeutic antibodies that block the actions of TNFα, IL-1β, or IL-6 are very efficacious in downregulating inflammatory disease, but 30–50% of patients with RA are resistant to the antibodies [19]. Clinicians need new therapeutic agents for RA patients. Recently, TLR signaling pathways have been implicated in the progression of RA, especially in late stages of disease. The TLR4 signaling pathway is regarded to be more important than any other signaling pathway in the progression of RA [5]. Therefore, in this study, for the first time, TLR4 inhibitor, TAK-242 (resatorvid), was tested for the potential to be developed as a new efficient therapeutic agent against RA. TAK-242 was selected from the Takeda chemical library as a therapeutic agent for sepsis treatment [20]. It exhibited the most potent suppressive activity for the production of not only NO but also inflammatory cytokines, including tumor necrosis factor-alpha (TNF-alpha) and interleukin-6 (IL-6), induced by LPS-stimulated mouse macrophages. In addition, its mode of action was already elucidated; it binds to Cyc747 of the intracellular domain of TLR4 and so interferes with recruiting of adaptor molecules such as TIRAP or TRAM to TLR4 [10]. In addition, much is known about its toxicity and clinical use [21]. Based on the concept of drug repositioning, we evaluated whether TAK-242 showed the potential to be cost-effectively developed as a remedy for rheumatoid arthritis or to control the progression of RA.

In this study, TAK-242 significantly inhibited the production of IL-6, IL-8, MMP-1, and VEGF in the LPS-stimulated human synovial cell line MH7A and primary human FLS. It also showed specific inhibition of IL-6 and IL-8 gene expression in LPS- or poly(I:C)-stimulated FLS, but not in IL-1β-stimulated FLS. One of the molecular mechanisms by which TAK-242 was thought to inhibit gene expression was by inhibiting the migration of NF-κB into the nucleus. The fact that TAK-242 inhibits NF-κB activation induced by LPS has been already demonstrated in HEK293 cells [11]. In contrast, in this study, we showed that it inhibited the production of IL-6 and IL-8 activated by poly(I:C), which stimulates the TLR3 signaling pathway. Among TLRs, TLR4 activation would be more involved in CFA-induced arthritis rats [22–24], making the AIA model a good choice to test the anti-arthritic effect of TAK-242.

As for the molecular mechanisms by which TAK-242 exerts anti-inflammatory effects, we showed that it inhibits the increased expression of NF-κB and AP-1 in the joints of AIA rats. In our AIA rat model, the expression of both transcription factors also significantly increased in joint tissue. Other study previously showed that the expression of these two transcription factors is not constitutive and significantly increases in a collagen-induced arthritis (CIA) mice model [25]. Further, the TLR-NF-κB pathway is known to be involved in triggering the inflammatory and joint destructive process in RA [26]. These things indicate that NF-κB and AP-1 are important factors in arthritis induction in the AIA rat model, and TAK-242 effectively decreased the expression of both transcription factors. The treatment dose of TAK-242 (3 or 5 mg/kg) was decided based on the previous studies [27, 28].

The therapeutic effect of TAK-242 has been tested in other disease animal models. It inhibited serum cytokine levels and improved survival in a mouse sepsis model when it was co-administered with antibiotics [29], making it a promising therapeutic agent for sepsis. However, it failed in phase III clinical human trials because it did not improve signs of sepsis after 28 days of administration [30]. Another TLR4 antagonist, eritoran, also failed in clinical studies of severe sepsis [31]. The TLR4 signaling pathway can be stimulated by various factors, including activation during diabetes. Inhibition of TLR4 signaling pathways by CLI-095 attenuates high glucose-induced reactive oxygen species (ROS) production and NF-κB activity in vascular smooth muscle cells (VSMCs) and improves vascular dysfunction and oxidative stress in diabetic rats [32]. In contrast to its beneficial effect, local peripheral TLR4 blockade seems to aggravate nociceptive pain thresholds in CFA inflammation. It has been suggested that TLR4 antagonists as new treatments for sepsis and neuropathic pain might unexpectedly transiently enhance pain by impairing peripheral opioid analgesia, because TLR4 activation of macrophages triggers opioid peptide release and thereby stimulates peripheral opioid-dependent anti-nociception [33]. Nevertheless, new TLR4 inhibitors have been invented and applied to diseases such as transplant rejection, chronic pain and nociception, and TLR4-mediated inflammation [21]. Although several preclinical trials using new TLRs inhibitors have been successfully performed and patented, clinical success remains limited. Thus, TLR-mediated immune responses need to be further clarified to treat immune diseases effectively through proper regulation of the TLR signaling pathway. Through a greater understanding of TLR biology, new methods or agents can be developed to downregulate TLR signaling pathways in different disease conditions.

## Conclusions

TAK-242 exerts an anti-arthritic effect in AIA rats through the inhibition of NF-κB and AP-1 activation. In this study, we demonstrated the anti-arthritic effect of TAK-242 in AIA rats for the first time. TAK-242 has the potential to be developed as a therapeutic agent against rheumatoid arthritis, even though the therapeutic effect on our arthritis animal model could not be extended to humans. Other inhibitors of TLR4 or other TLRs need to be tested as potential therapeutic agents against RA. Given that patient responsiveness to drugs is quite variable, and that some patients may be resistant to a drug, TLR inhibitors represent another option for some patients as therapeutic medications. Further studies of TLR inhibitors are required, particularly given the potential for cost-effective development as a remedy.

## Data Availability

Please contact author for data requests.

## Abbreviations

AIA: Adjuvant-induced arthritis
ANOVA: Analysis of variance
CFA: Complete Freund’s adjuvant
FLS: Fibroblast-like synoviocytes
LPS: Lipopolysaccharide
RA: Rheumatoid arthritis
TLR: Toll-like receptor
